# A Portable Tunnel Electromagnetic Impulse Shock Source

**DOI:** 10.3390/s23094213

**Published:** 2023-04-23

**Authors:** Yuning Hu, Wei Wang, Wei Liu, Zihan Chen, Jie Zhou, Zhihong Fu

**Affiliations:** 1 State Key Laboratory of Power Transmission Equipment and System Security and New Technology, Chongqing University, No. 174 Shazhengjie, Chongqing 400044, China; 2 School of Electrical Engineering, Chongqing University, No. 174 Shazhengjie, Chongqing 400044, China

**Keywords:** tunnel seismic prediction, artificial seismic source, electromagnetic acceleration, pulse power

## Abstract

Early seismic prediction in tunnels has become a necessary task to prevent construction risks. The type and performance of the seismic source are crucial factors that affect its efficiency and prediction accuracy. Among the existing main types of tunnel seismic sources, both explosive and spark sources require pre-drilling and pose safety hazards. In addition, explosive sources pose a risk of damage to tunnel structure walls. Spark sources must be used in a water medium. Artificial hammer sources have weak energy and short prediction distance. This paper proposes a portable tunnel electromagnetic impulse shock source that overcomes the deficiencies of the aforementioned seismic sources. This paper elaborates on the design and working principle of the electromagnetic impulse shock source, and analyzes the electromagnetic force exerted on the hammer body during acceleration. Through finite element simulation, this paper analyzes the multi-physical field changes of the entire electromagnetic acceleration system. Meanwhile, combining experimental testing, the design parameters and energy of the seismic source are optimized to finally design a portable electromagnetic impulse shock source. The engineering measurement data results show that the seismic source can stably generate an output energy of 1500 J, and the overall weight of the seismic source does not exceed 40 kg, with a single shot time less than 30 s and an effective prediction distance over 100 m. This work has significant practical value and superiority.

## 1. Introduction

Tunnel seismic early warning can provide information about adverse geological formations ahead of the heading face within a distance of 100–120 m. Combined with geological data, it can accurately determine the location of abnormal formations such as karst caves, fault zones, and weak interlayers ahead of the heading face. According to the classification of observation systems, tunnel seismic prediction methods mainly include tunnel seismic prediction (TSP) [1,2,3], comprehensive seismic imaging systems [4,5,6], horizontal seismic profile [7,8], acoustic soft soil foundation detection [9], tunnel seismic tomography [10,11,12,13], tunnel seismic while drilling method [14], and three-dimensional true reflection tomography (TRT) [15,16,17,18]. The seismic sources used in these methods are mainly divided into explosive and non-explosive types. Explosive seismic sources generally use small explosives (30 g to 100 g), which have strong energy and can produce seismic waves with good pulse properties [19]. The collected signals have a wide frequency band and high signal-to-noise ratio, and the prediction distance can reach 100 m to 120 m. However, the use of explosive seismic sources requires pre-drilling, which not only increases the additional workload but may also damage the tunnel wall. At present, most engineering fields use TSP observation systems, which can only stimulate 24 shots on one side of the tunnel wall, and the limitation of the number of shots will affect the accuracy of data processing.

Non-explosive sources mainly include the following: ① Impact hammer source. This is composed of a hammer body and a cutting board. The hammer body can be a hanging hammer or an artificial sledgehammer. The free-falling body is used to accelerate the hammer body, which can excite high-frequency elastic waves. The sledgehammer source does not need to drill in advance, and the shooting cost is low. It can be shot multiple times at the same location to enhance the signal-to-noise ratio. The sledgehammer can be hammered on both sides of the tunnel wall, and the number of hammers can be more than 24. However, the energy of the sledgehammer source is low, the collected signal frequency band is narrow, and the signal is vulnerable to mechanical noise interference. The prediction distance can only reach 80 m. In terms of signal-to-noise ratio, bandwidth and prediction distance, the explosive source is superior to the sledgehammer source. In terms of construction efficiency, safety and flexibility, the sledgehammer source is superior to the explosive source. ② Electric spark source. The capacitor is used to store energy, and the energy is released instantaneously when the discharge electrode breaks through the water medium, producing the effect of an explosion and then triggering the seismic wave. This kind of source has high requirements for switching. The discharge electrode needs to be placed in water before it can be excited [20]. ③ Swept frequency source. Seismic waves can be generated by continuous vibration, and the frequency can be controlled. Although the energy of a single excitation is small, it can still detect a relatively deep range after the superposition of multiple seismic waves. It is generally vehicle-mounted, bulky and expensive, and its use in mountainous areas and tunnels is limited [21,22,23]. ④ Shock source. This kind of source is very similar to the seismic wave generated by the explosive source. One shot can obtain the seismic wave with good pulse property and high energy. The common ones are high-pressure gas shock source [24] and mechanical shock source. The mechanical shock source uses the spring to accelerate the hammer body to obtain kinetic energy. It is not convenient to reset the hammer body, and the energy is not easy to increase. The high-pressure gas shock source uses high-pressure gas to accelerate the hammer to obtain kinetic energy. Although the energy is high, the design is complex and requires the design of energy storage and energy release devices. The volume is too large.

The geologic examination of urban roads, tunnels and underground space requires a small impact on the environment; the explosive source has strong destructive power, and belongs to controlled goods. The risk factor is high. Obviously, it is not the best plan for these scenarios. Although the effect of an electric spark source is the closest to that of an explosive source in the process of excitation, it needs to be in the water to complete the excitation, and the large capacity electric spark source is bulky and costly. Due to the limited frequency points and low resolution, it is difficult to meet the needs of refined exploration with a swept frequency source. The impact hammer source is characterized by wide frequency band and high construction efficiency. It is an ideal urban seismic survey source, especially the electromagnetic shock source. It has the characteristics of portability, high construction efficiency and good excitation signal, and has a good application prospect. However, there are few reports on the research of electromagnetic shock sources at home and abroad at present.

The portable electromagnetic shock source proposed in this paper has many advantages, such as lightweight, small size, convenient operation, convenient transportation, sufficient excitation energy, high construction efficiency, rich excitation signals, etc. This kind of source uses the principle of a magneto-resistive electromagnetic transmitter to drive the ferromagnetic projectile into the measured body through an accurate current pulse, and excite the elastic wave, thus realizing seismic exploration. Due to its characteristic of multi-directional excitation, it can be applied in different exploration environments, such as wild mountains, urban roads, urban underground space, etc. In addition, compared with the traditional explosive source, the electromagnetic shock source causes less damage to the environment in the exploration process, and its operation is safe and reliable, so it has a better application prospect.

## 2. Principle of Electromagnetic Source

The electromagnetic shock source uses the principle of a magneto resistive electromagnetic transmitter to accelerate the hammer. According to the principle of minimum magnetic resistance, the magnetic flux always tends to pass through the path of minimum magnetic resistance. Therefore, there is an electromagnetic force of mutual attraction between the drive coil and the hammer with high permeability. It can also be understood that the current magnetized by the ferromagnetic hammer is the same as the current direction of the drive coil, so it is subject to the electromagnetic force of mutual attraction [25].

### 2.1. Circuit Design

**Figure 1:**
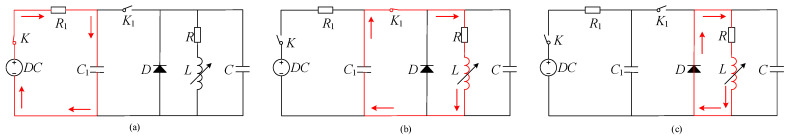
Principle of capacitor power supply circuit. (**a**) Capacitor charging, (**b**) Capacitor discharge, (**c**) Afterflow process.

At present, there are two common energy storage units for electromagnetic acceleration: capacitor and battery. The circuit schematic diagram of a capacitor as an energy storage unit is shown in Figure 1.

With the capacitor as the energy storage unit, the workflow includes capacitor charging, capacitor discharge and afterflow. In Figure 1, is charging power supply, K is a capacitor charging circuit switch, $R_{1}$ is a current limiting resistor, $C_{1}$ is an energy storage capacitor, $K_{1}$ is a capacitor discharge circuit switch, D is a freewheeling diode, R is coil resistance, L is coil inductance, and C is coil distributed capacitance. Figure 1a shows the capacitor charging process. The DC high-voltage power supply charges the high-voltage energy storage pulse capacitor $C_{1}$ through the current-limiting resistor. After charging, the switch K is disconnected and one waits for the discharge signal. After charging and receiving the discharge signal, the switch $K_{1}$ is closed to form a discharge conduction circuit. The coil has current flowing through it to generate a magnetic field to accelerate the hammer in the moving cavity. At the same time, the coil acts as an inductor to store a certain amount of energy during the discharge process, which is shown in Figure 1b. Then, as shown in Figure 1c, the circuit converts to the afterflow process. During the discharge process, the inductor accumulates energy. At the moment of turning off $K_{1}$, the inductor forms a freewheeling circuit through the freewheeling diode, and consumes the energy stored during the discharge process through the coil resistance.

**Figure 2:**
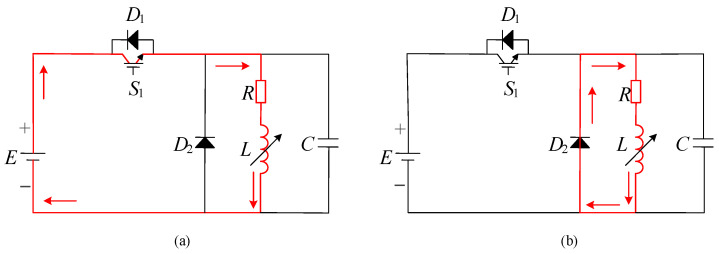
Battery power supply circuit principle: (**a**) Discharge process, (**b**) Afterflow process.

The schematic diagram of the working circuit of the multidirectional electromagnetic shock source is shown in Figure 2. The method of direct battery power supply is used to regulate the power-on time by controlling the on-off of the IGBT. E is the energy storage battery, $S_{1}$ is the control switch IGBT, R is the coil resistance, L is the coil inductance, C is the coil distributed capacitance, and $D_{2}$ is the freewheeling diode. Figure 2a describes the circuit discharge process. At this time, the IGBT is closed, the circuit is connected, and the battery is directly powered to form a conduction circuit. The coil has current flowing through it to form a magnetic field to accelerate the hammer in the moving cavity. At the same time, the coil acts as an inductor to store a certain amount of energy during the discharge process. As shown in Figure 2b, when turning off the IGBT, the inductor forms an afterflow through the freewheeling diode $D_{2}$, and consumes the energy stored during the discharge process through the coil resistance. The single working process of the electromagnetic source consists of the discharge process and afterflow process.

**Figure 3:**
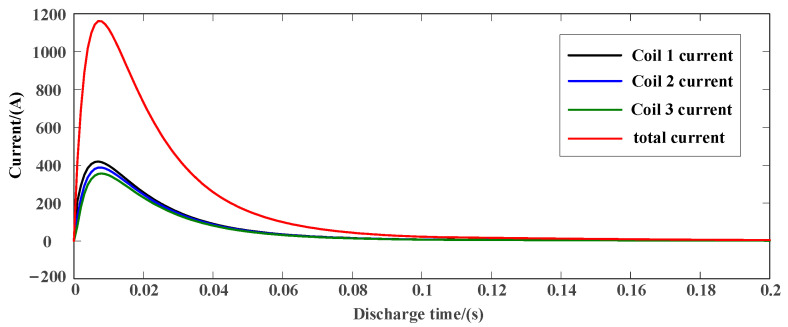
Capacitor discharge current waveform.

**Figure 4:**
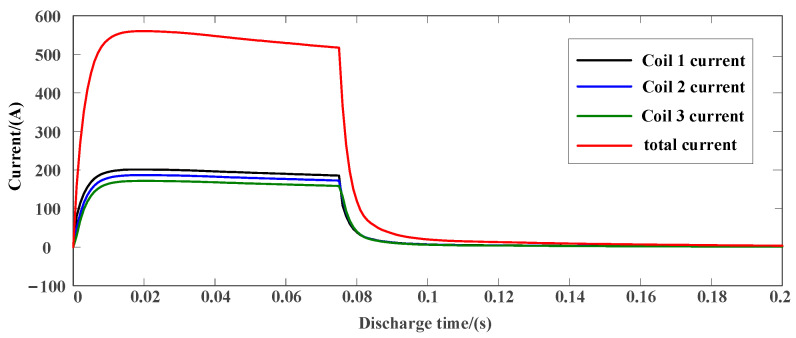
Battery discharge current waveform.

As shown in Figure 3 and Figure 4, the discharge current waveform of the capacitor and the discharge current waveform of the battery are, respectively, under the condition of the same discharge energy of the capacitor and the battery. It can be seen from the discharge current waveform that the capacitor discharge current pulse is high, but the current changes greatly, and the discharge current is unstable. Although the electromagnetic force generated at the maximum current is greater, its duration is short, the acceleration process is unstable, and the final hammer speed is only 6.9 m/s. The discharge current waveform of the battery is similar to the square wave, the current is more stable, the effective discharge current duration is long, the stable acceleration time is long, and the final hammer speed can reach 12 m/s. The multidirectional electromagnetic shock source adopts the battery direct power supply mode, which is not only simpler in the circuit, but also shortens the working cycle, and can make the hammer obtain higher kinetic energy under the same energy storage condition.

In the discharge circuit directly powered by batteries, IGBT is used as the discharge switch, with a maximum current of 600 A. Through actual testing, the resistance value of the discharge circuit is approximately 0.32 Ω, so the supply voltage is controlled at 175 V, and the discharge current is 550 A.

### 2.2. Structural Design

**Figure 5:**
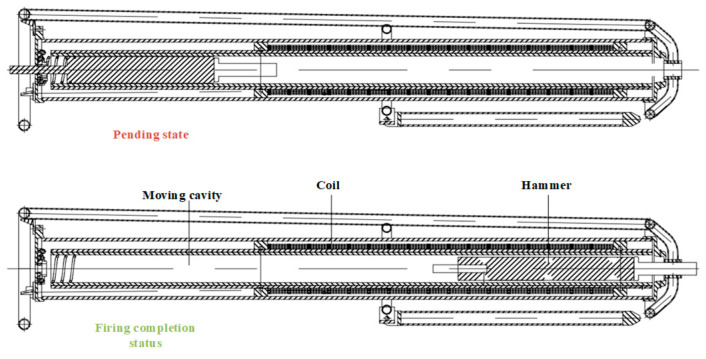
Section views of the structure of the initial and final state of the electromagnetic shock tube.

The structural profile of the initial and final states of the electromagnetic shock source is shown in Figure 5. The electromagnetic hammer part mainly includes the electromagnetic accelerator, hammer body, hammer body self-locking/releasing device, protective support and other accessories. When the hammer is going to be excited, the hammer is locked by the self-locking/releasing device. When the operator gives the excitation signal, the hammer is released, and the main circuit switch is triggered to drive the electromagnetic accelerator with pulse power. Under the pulse magnetic field of the electromagnetic accelerator, the hammer is accelerated instantaneously and impacted forward until hitting the check position, and the seismic wave will be transmitted to the geological interior. After that, the hammer will be rebounded to the initial position and self-locks, waiting for the next excitation signal.

**Figure 6:**
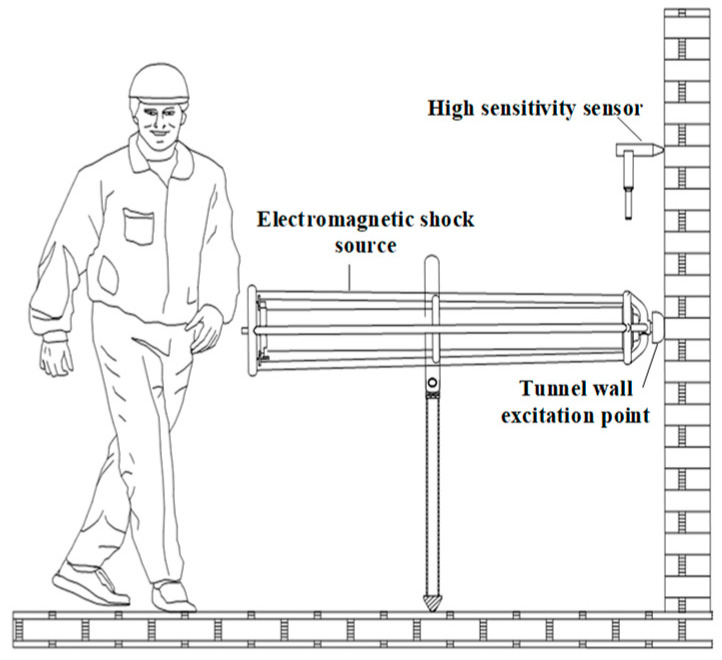
The detection method of portable electromagnetic shock tube.

The detection method of a multiple-direction portable electromagnetic shock source is shown in Figure 6. In reality, the electromagnetic shock source is applicable to any scene since it can be excited in any direction. For example, in tunnel seismic detection, it can be laterally excited, when used for detection of urban roads or urban underground space, the electromagnetic shock source is vertically excited.

### 2.3. Energy Analysis

The law of conservation of energy can be expressed as

(1) $$\Delta W_{i}=\Delta W_{m}+\Delta W_{k}+\Delta W_{loss}$$

where $\Delta W_{i}$ is the increase in energy input by the energy storage unit to the coil. $\Delta W_{m}$ denotes the increase in energy stored by the coil, that is, the increase in magnetic field energy in the whole space area. $\Delta W_{k}$ means the increase in hammer kinetic energy, and $\Delta W_{loss}$ is the energy lost by the source system.

The energy loss of the system mainly includes the heat loss of the loop resistance, the friction loss between the hammer body and the conduit, the hysteresis loss in the hammer body, and the eddy current loss. Among them, friction loss, hysteresis loss, and eddy current loss are relatively small; the main part is the heat loss of coil resistance.

In order to find out the parameters related to the electromagnetic force qualitatively, the decrease in magnetic field energy is assumed to be the increase in hammer kinetic energy, that is

(2) $$\Delta W_{m}=-\Delta W_{k}$$

The magnetic field energy in the space area is

(3) $$W_{m}=\frac{1}{2}\int_{V_{1}}\vec{H}\cdot \vec{B}dV+\frac{1}{2}\int_{V_{2}}\vec{H}\cdot \vec{B}dV$$

where $V_{1}$ is the volume occupied by the hammer, and $V_{2}$ is the volume corresponding to the air. The magnetic medium represented by $\vec{B}$ can be calculated as

(4) $$\vec{B}=\mu \vec{H}$$

And μ is the permeability of the magnetic medium. The mode of the magnetic field strength $\left|\vec{H}\right|$ can be expressed as a function of parameters *z* and *i*:

(5) $$\left|\vec{H}\right|=f(z,i)$$

where *z* is the distance between the hammer body and the coil, and *i* is the coil current. In a very short time of *t*, the hammer body moves from coordinate $\left(z_{0},t_{0}\right)$ to $\left(z_{t},t_{t}\right)$, and the increase in magnetic field energy is

(6) $$\Delta W_{m}=W_{m(z_{t},t_{t})}-W_{m(z_{0},t_{0})}$$

In addition, the kinematics formula is written as

(7) $$\Delta E_{k}=F\cdot d_{z}$$

Combined with Equations (2)–(7), the electromagnetic force *F* can be defined as follows:

(8) $$F=\frac{\mu_{Fe}[\int_{V_{1}}f^{2}(z_{0},i_{0})dV-\int_{V_{1}}f^{2}(z_{t},i_{t})dV]}{2dz}+\frac{\mu_{0}[\int_{V_{2}}f^{2}(z_{0},i_{0})dV-\int_{V_{2}}f^{2}(z_{t},i_{t})dV]}{2dz}$$

where $\mu_{Fe}$ is the permeability in the hammer and $\mu_{0}$ is the permeability in the air.

To sum up, the electromagnetic force is influenced by the hammer position, magnetization curve of the hammer and parameters in the discharge circuit. If the current discharge time is too short and the current peak is high, the magnetic induction intensity is easy to saturate. However, if the current discharge time is too long, reverse pumping will occur during the acceleration process. In both cases, the hammer body cannot obtain higher speed. Therefore, it is necessary to optimize the relevant influence parameters to match the discharge time with the hammer movement time as much as possible so that the hammer can obtain more kinetic energy.

## 3. Analysis of Simulation and Measured Results

### 3.1. Finite Element Simulation

**Figure 7:**
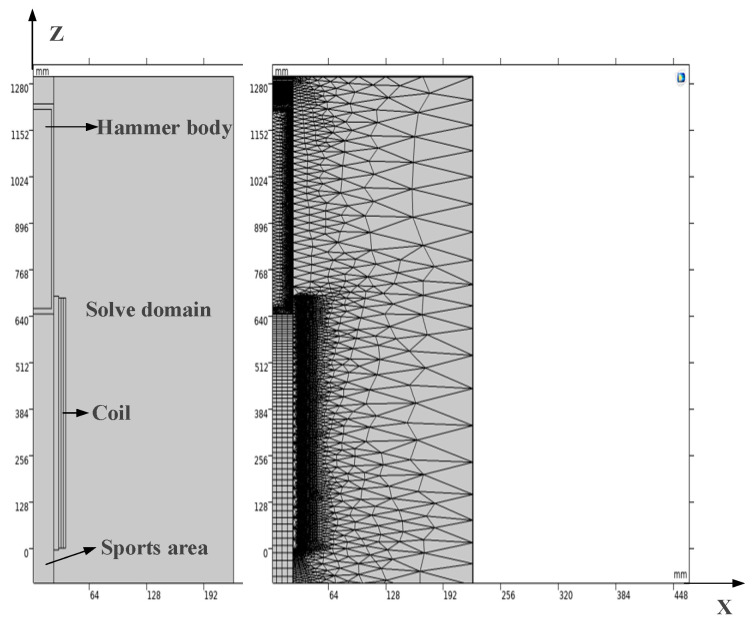
Two-dimensional simulation model of electromagnetic source.

In this paper, COMSOL finite element simulation software is used to carry out the 2D simulation calculation of the electromagnetic launch process. Since the multi-azimuth electromagnetic shock source is an axially symmetric device, when analyzing the influence of parameters such as the power-on time and the initial position of the hammer on the launch energy, it can be solved by 2D finite element simulation. As shown in Figure 7, a 2D simulation model of the z-axis symmetry is established according to the specific size parameters in Table 1.

**Figure 8:**
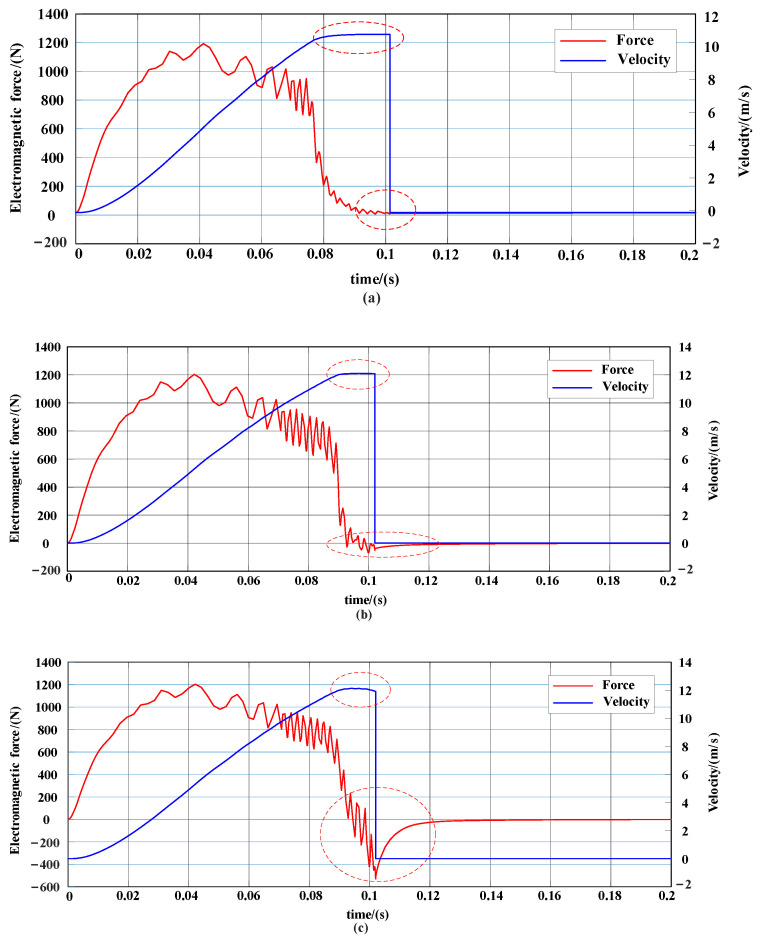
Variation curve of electromagnetic force and speed under different power-on times. (**a**) Electromagnetic force and speed change curve with power-on time of 80 ms. (**b**) Electromagnetic force and speed change curve with power-on time of 90 ms. (**c**) Electromagnetic force and speed change curve with power-on time of 100 ms. The red dotted circle respectively represent the trend of speed change and the presence or absence of reverse electromagnetic force. When the power on time is too long, the speed in the red dashed lines tends to decrease, and the reverse electromagnetic force is relatively large.

According to the principle of the magneto resistive electromagnetic transmitter, when the central position of the hammer body coincides with the central position of the coil, if the hammer body continues to move forward, the direction of the electromagnetic force will be reversed, so the power-on time needs to be determined according to the position of the hammer body. If the power-on time is too short, the acceleration process is too short to reach the maximum speed. If the power-on time is too long, there will be a reverse electromagnetic force to slow down the hammer body, and the speed cannot reach the optimum. Thus, there must be an optimal solution for the power-on time. The initial position indicates the length of the hammer entering the coil. On the premise that the initial position is 5 cm, the change of the electromagnetic force and speed is observed by simulation when the power-on time is 80 ms, 90 ms, and 100 ms, as shown in Figure 8a–c.

As shown in Figure 8a, when the power-on time is 80 ms, the discharge time is short, and the IGBT has been turned off before the hammer moves to the center position coincidence point. At this time, no reverse electromagnetic force is generated, but the hammer does not reach the maximum speed. Figure 8b shows that the power-on time is 90 ms, at which time the hammer body has moved to the vicinity of the central position coincidence point, and has generated a small reverse electromagnetic force, and the hammer body speed has not significantly decreased. Figure 8c reflects that the power-on time is 100 ms. In this setting, the hammer body has passed the center position coincidence point, generating a reverse electromagnetic force with a peak value of 500 N, and the hammer body speed has decreased significantly.

From the simulation results, it can be seen that the power-on time is an important factor affecting the final velocity of the hammer body, and has certain regularity, which is closely related to the real-time motion position of the hammer body. At the same time, the hammer initial position is also an important parameter which affects the final speed. The simulation results provide theoretical support and guide the range of parameters in the actual measurement.

### 3.2. Experimental Test and Result Analysis

**Figure 9:**
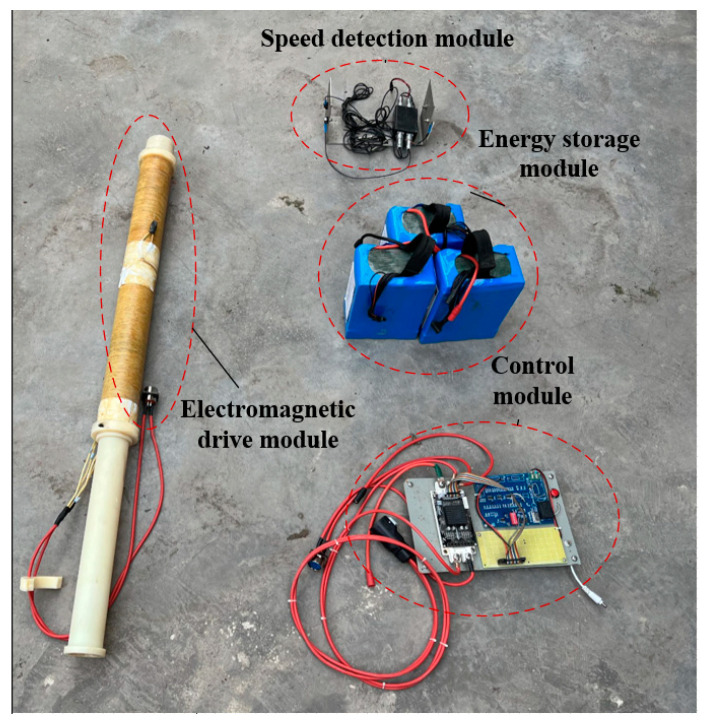
Energy test system. The red dashed circles represent the various components of the energy testing system, including electromagnetic drive module, energy storage module, speed detection module, and control module.

**Figure 10:**
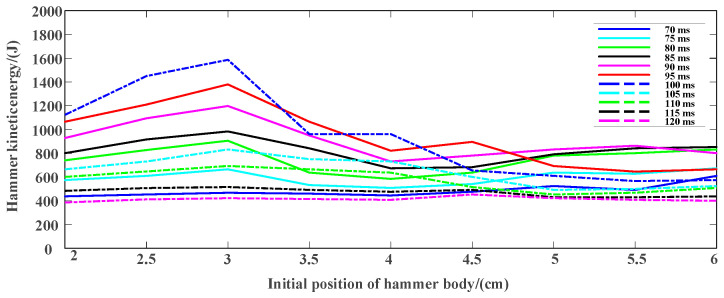
Kinetic energy of the hammer at different initial positions.

**Figure 11:**
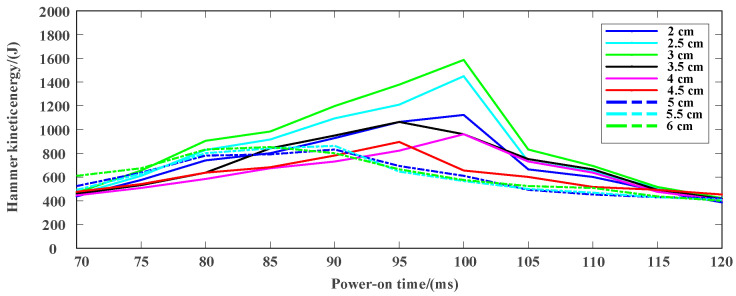
Kinetic energy of the hammer at different power-on times.

**Figure 12:**
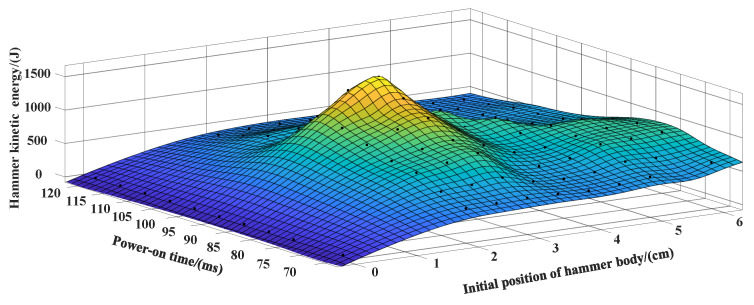
Three-dimensional diagram of experimental data.

The experimental platform is shown in Figure 9, mainly including the energy storage module, control module, electromagnetic drive module, and speed detection module based on a photoelectric gate. The speed measurement module consists of two sets of photoelectric gates with a distance of 5 cm. When both sets of photoelectric gates are blocked or not blocked, the output is at a low level. When there is only one set of photoelectric gates blocked, the output is at a high level. By measuring the time it takes for the hammer to block the first set of photoelectric gates and completely block the two sets of photoelectric gates (corresponding to the high-level duration of the waveform measured by the oscilloscope), the emission velocity of the hammer can be calculated from $v=\frac{s}{t}$. Then, the kinetic energy of the hammer body can be calculated using the kinetic energy calculation formula. The results of Figure 10, Figure 11 and Figure 12 in this article are all calculated using this method.

The experimental object is a magneto-resistive electromagnetic transmitter with 1200 turns of winding and three groups of coils in parallel. Ten samples under different power-on times are tested at the initial position of each specific hammer. In order to reduce the loss caused by resistance heating, the diameter of the moving cavity shall be reduced as far as possible within the scope of size requirements, and the winding length shall be reduced, so as to reduce the internal resistance and weight of the copper wire.

Based on the requirements of the actual working conditions, the total length of the magneto resistive coil transmitter is about 1.2 m, and the power-on time and the initial position of the hammer are preliminarily screened by COMSOL simulation software. The specific experimental parameters are shown in Table 1. Taking the power-on time and the initial position as the main parameters to be optimized while fixing other parameters, the actual measurement and analysis are carried out and the final kinetic energy is compared. The experimental results are shown in Figure 10, Figure 11 and Figure 12.

According to the results in Figure 10 and Figure 11, it can be seen that the kinetic energy range of the hammer body is 400–1600 J, and different power-on time as well as initial position have great influence on the final kinetic energy of the hammer body. In Figure 12, when the power-on time is 100 ms, the hammer has the maximum kinetic energy with the initial position of about 3 cm.

As shown in Figure 10, the power-on time is short or long, such as 70 ms, 115 ms and 120 ms, the change of initial position has no obvious impact on the kinetic energy of the hammer. When the power-on time is short, the power supply is stopped before the electromagnetic force reverses, resulting in the end speed not reaching the optimum. The power supply is still on after the electromagnetic force reverses for a long time. The reverse electromagnetic force causes the hammer body to decelerate, so the end speed is not the maximum. In other power-on times, the change of the initial position has a significant impact on the kinetic energy of the hammer. The initial position is 3 cm, with the largest kinetic energy. On further analysis, as shown in Figure 11, the initial position remains unchanged, the power-on time increases gradually, and the kinetic energy of the hammer increases first and then decreases. Whether the initial position is small or large, and even if the power supply is interrupted when the hammer moves to the reverse of the electromagnetic force, the kinetic energy is small. One of the critical conditions is that the initial position is 0. At this time, the electromagnetic force is 0 at the moment of power-on, the initial velocity is 0, and the kinetic energy is 0. Another critical condition is that the initial position is the coincidence point of the center line of the hammer body and the center line of the coil. The resultant force of the electromagnetic force on the hammer body is 0 at the moment of power-on, the initial speed is 0, and the kinetic energy is 0. Therefore, if the initial position is too large or too small, the kinetic energy of the hammer will be reduced. To sum up, there must be an optimal parameter solution within the selected power-on time and initial position range.

**Figure 13:**
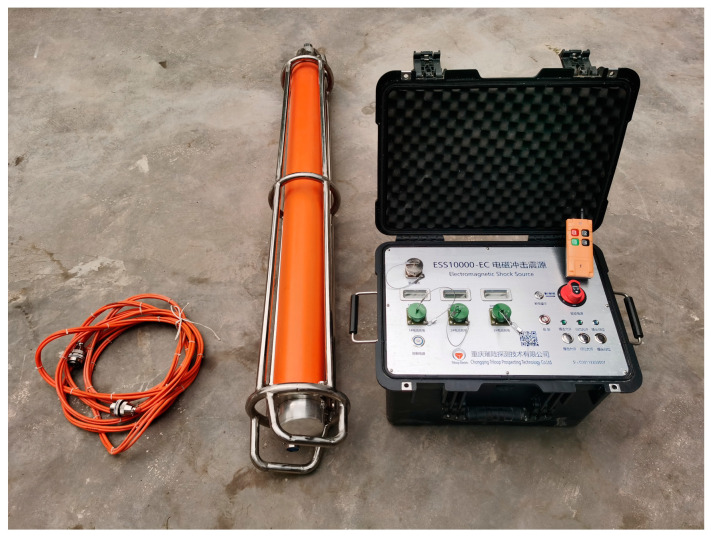
Tunnel electromagnetic shock source.

Based on the simulation results and the measured results, the initial position of the hammer body is determined to be 3 cm and the power-on time is 100 ms as the optimal solution of the parameters under the condition that the size of the electromagnetic source is required to be fixed under specific working conditions. The developed multidirectional tunnel electromagnetic shock source is shown in Figure 13.

## 4. Engineering Application

The measured tunnel is a tunnel in southwest China. The comparative experiments of the sledgehammer source, electromagnetic source and explosive source were carried out in the tunnel, and the advance prediction of the explosive source in the tunnel was also completed. The three sources were shot at the same detection position using the conventional TSP observation system.

The electromagnetic source work lasted about half an hour from the preparation to the end, and the explosive source work lasted nearly two hours from the preparation to the end. With the explosive source, there is a risk of data loss due to a bad channel.

### 4.1. On-Site Data Acquisition of Electromagnetic Source

**Figure 14:**
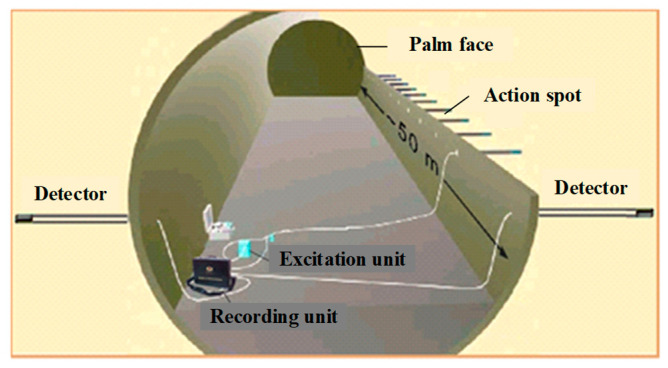
Schematic of the tunnel seismic prediction.

**Figure 15:**
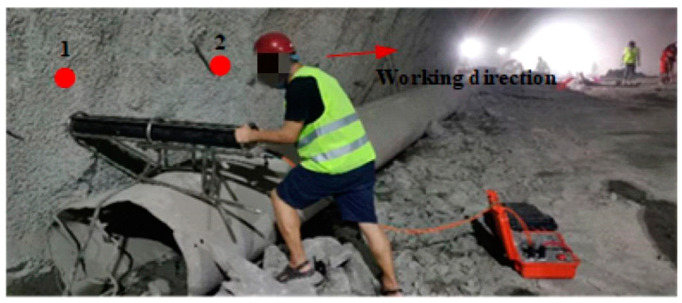
Working diagram of tunnel electromagnetic shock source.

In the process of tunnel seismic advance prediction, 24 shots were hammered on one side of the tunnel wall. The distance between the first shot and the palm face was 15 m, the distance between shots was 1m, and the distance between the last shot and the geophone was 10 m. The data acquisition sampling interval is 41.7 μs, and the sampling length is 167 ms. Figure 14 is the schematic diagram of tunnel seismic advance prediction, and the working site is shown in Figure 15.

### 4.2. Data Processing and Interpretation

**Figure 16:**
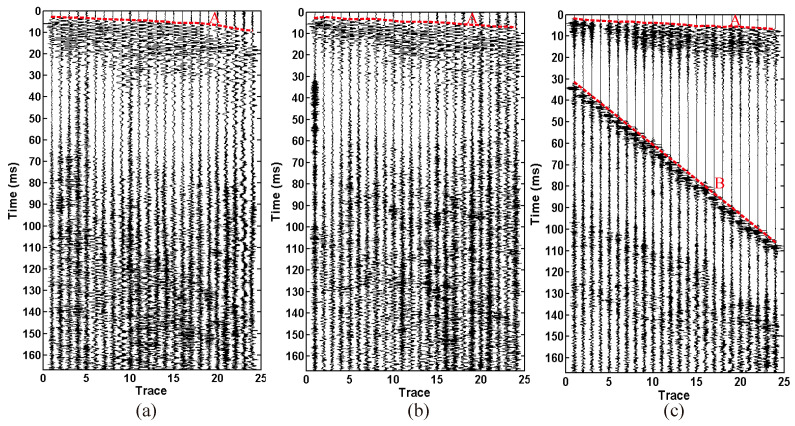
X-component original seismic record. (**a**) 24 pound sledgehammer. (**b**) Electromagnetic shock source. (**c**) explosive source. A is a direct wave and B is a sound wave.

As shown in Figure 16, the original seismic records of three source X components are shown. A is a direct wave and B is a sound wave. The acoustic energy in the seismic records of the sledgehammer source and the electromagnetic source is weak, while the acoustic energy in the seismic records of the explosive source is strong. During the data acquisition of the sledgehammer source and the electromagnetic source, the random noise in the seismic records is relatively strong due to the influence of large mechanical operations in the tunnel (Figure 16a,b).

**Figure 17:**
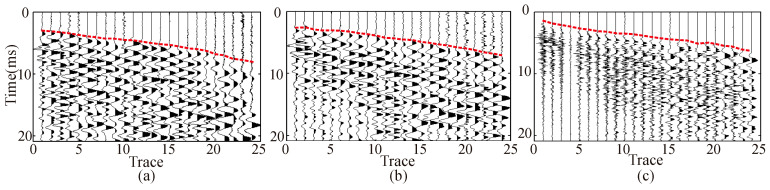
Amplified display of X-component original seismic record. (**a**) 24 pound sledgehammer. (**b**) Electromagnetic shock source. (**c**) explosive source. The red dashed line represents the first arrival of the wave.

Figure 17 is an enlarged display of the 0 ms to 20 ms seismic records in Figure 16. The first arrival time of the direct wave is easy to identify. According to the first arrival picked up in Figure 17, the longitudinal wave velocities of the surrounding rock are calculated by the least square fitting method as 5995 m/s, 5876 m/s, and 5733 m/s. Theoretically, the velocity of the surrounding rock calculated from the first break of the explosive source is closest to the real velocity of the surrounding rock, and the estimation accuracy of the velocity of the surrounding rock from the electromagnetic source is closer to that of the explosive source.

**Figure 18:**
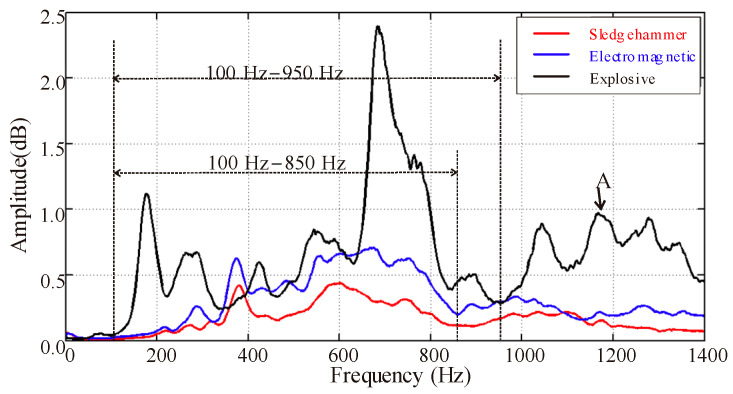
Spectrum analysis.

As shown in Figure 18, the spectrum analysis results show that the main frequency range of explosive source data is 100 Hz to 950 Hz, and the signal with frequency greater than 1000 Hz is mainly acoustic interference. The main frequency range of data from the sledgehammer source and the electromagnetic source is 100 Hz to 850 Hz. The peak energy of the spectrum curve shows that the explosive source has the highest energy and the sledgehammer source has the lowest energy.

**Figure 19:**
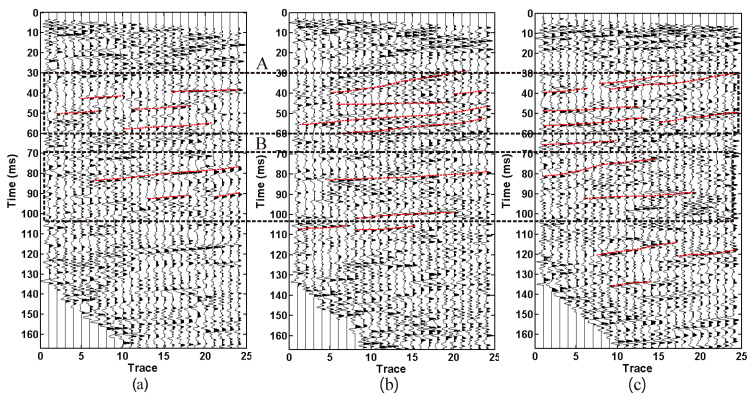
Data processing results. (**a**) 24 pound sledgehammer. (**b**) Electromagnetic shock source. (**c**) explosive source. The red line is identified as the reflection wave from the leading direction.

Figure 19 shows the three source data processing results. In order to facilitate the comparison of the later results, the filtering parameters in the bandpass filtering processing are unified from 80 Hz to 800 Hz. A waveform with a continuous in-phase axis and strong energy is considered as a reflection wave from the leading direction. The red line in Figure 19 is identified as the reflection wave from the leading direction. The reflection wave of the three sources from 30 ms to 60 ms is relatively similar, while the reflection wave of the electromagnetic source and the explosive source from 70 ms to 105 ms is relatively similar, but the reflection wave of the sledgehammer source is quite different. The number of reflected waves from the sledgehammer source in the 70 ms to 105 ms section is small and the continuity is low.

**Figure 20:**
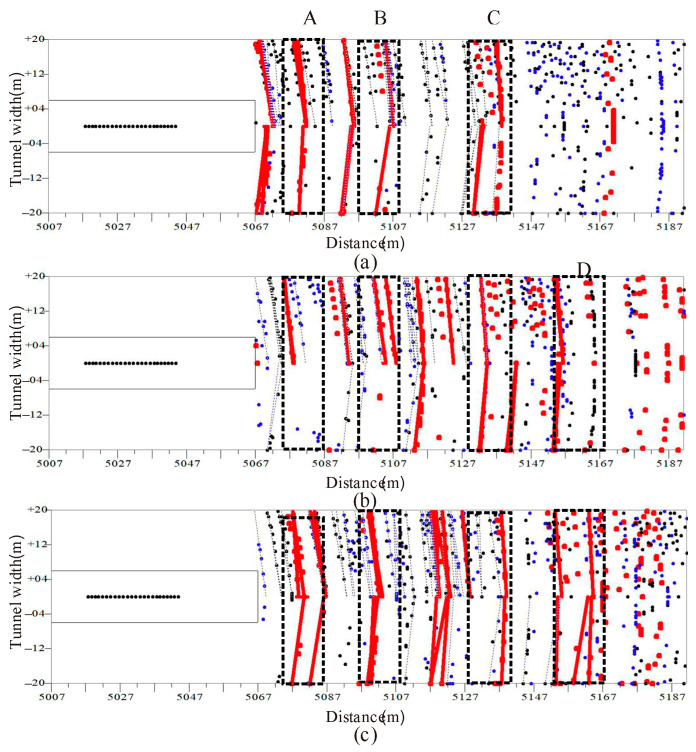
Forecast Interpretation Chart. (**a**) 24 pound sledgehammer. (**b**) Electromagnetic shock source. (**c**) explosive source. The red line and red dot represent the position of the strong impedance interface extracted from P-wave migration data, the blue line and blue dot represent the position of the strong impedance interface extracted from SV wave migration data, and the black line and black dot represent the position of the strong impedance interface extracted from SH wave migration data. The red line, blue line, and black line are interfaces with relative energy greater than 20%, while the red dot, blue dot, and black dot are interfaces with relative energy between 20% and 50%.

As shown in Figure 20, the prediction interpretation diagram of the three sources is shown. The three source results have a strong reflection interface at mileage K5 + 076 to K5 + 087 (A), K5 + 097 to K5 + 108 (B), and K5 + 128 to K5 + 142 (C). When the prediction distance exceeds 70 m, there is no obvious strong reflection interface in the prediction interpretation map due to the low energy of the sledgehammer source. The electromagnetic source and explosive source have a strong reflection interface at the mileage K5 + 152 to K5 + 167, and the prediction distance reaches 100 m.

The data prediction distance of the 24-pound sledgehammer source can only reach 70 m, while the data prediction distance of the electromagnetic source and the explosive source can reach 100 m, and the prediction effect of the electromagnetic source is close to that of the explosive source.

The spatial resolution of the seismic source equipment is defined as one fourth of the length of the seismic wave being excited, and the wavelength of the seismic wave refers to the ratio of the surrounding rock velocity to the excitation frequency. The measured velocity of the surrounding rock in this article is about 5733 m/s, and the excitation frequency is 100–850 Hz. Therefore, the electromagnetic source equipment in this article has calculated that the maximum spatial resolution can reach 1.7 m.

## 5. Discussion and Conclusions

The multidirectional portable electromagnetic shock source designed in this paper is directly powered by batteries. Compared with the source powered by a capacitor charging and discharging, the circuit is simpler, the discharge efficiency is higher, and it has a more compact volume. After circuit optimization, the control is simplified, which has a higher fault tolerance rate, the acceleration process is more stable, and the kinetic energy is greater. In order to obtain sufficient energy, the optimal initial position and power-on time are obtained by combining the simulation analysis with measured results. Finally, the power-on time of 100 ms and the initial position of 3 cm are proved to produce the maximum excitation energy.

Compared with explosive sources, the electromagnetic source can repeatedly impact to ensure that there is no low signal-to-noise ratio signal, and the single tunnel test takes 25 min, greatly shortening the construction period. At the same time, its excitation effect is very close to the explosive source but has lower risk. As for the artificial sledgehammer source, the electromagnetic source has strong energy and a high signal-to-noise ratio of the reflected wave. Therefore, the electromagnetic shock source is a high-performance artificial source that can completely replace the explosive source in the tunnel advanced prediction engineering.

Considering the portability requirements of tunnel and ground environment construction operations, the multidirectional electromagnetic shock source needs to have the characteristics of small size, light weight and strong excitation energy. The simulation and actual test results show that the source excitation energy can reach 1500 J under the limit of limited physical size, which can fully meet the actual engineering application requirements. The total weight of the main parts is not more than 40 kg, which is small in size, simple in operation and portable in transportation, it can be conveniently applied to various geological exploration environments.

## Figures and Tables

**Table 1:** Energy test experimental parameters.

Parameter	Numerical Value
Number of winding turns	1200
Transmitter length	1.2 m
Discharge Voltage	175 V
Discharge Time (ms)	70, 80, 85, 90, 95, 100, 105, 110, 115, 120
Initial Position of Hammer Body (cm)	2, 3, 3.5, 4, 4.5, 5, 5.5, 6

## Data Availability

Not applicable.
